# Risk factors for death in chronic critical illness

**DOI:** 10.1186/2197-425X-3-S1-A449

**Published:** 2015-10-01

**Authors:** S Lokhandwala, B Escobar, A Chahin, N Mccague, M Ghassemi, M Feng, LA Celi

**Affiliations:** Harvard-MIT Division of Health Science and Technology, Laboratory of Computational Physiology, Cambridge, MA USA; Beth Israel Deaconess Medical Center, Internal Medicine, Boston, MA USA; Beth Israel Deaconess Medical Center, Infectious Disease, Boston, MA USA; Beth Israel Deaconess Medical Center, Pulmonary and Critical Care, Boston, MA USA

## Introduction

As early recognition, resuscitation and technological advancement in the treatment of critically ill patients have improved, efforts to understand patient outcomes after an acute period of illness are being undertaken. While it has been noted that one-year mortality among survivors of critical illness is extremely high, awareness is increasing regarding patients who, despite survival, remain “chronically critically ill”. This is important considering that patients who have short-term survival after ICU discharge have poor quality of life leading to death.

## Objectives

To determine among a cohort of ICU patients who survived >30 days factors that are associated with survival less than one year.

## Methods

We conducted a longitudinal, single center, retrospective cohort study of patients admitted to an intensive care unit at Beth Israel Deaconess Medical Center using the MIMIC database. Patients were included if they survived greater than 30 days post discharge and excluded if they were known to have advanced cancer. The 1-year survivors and non-survivors were compared using the Wilcoxon rank sum test for continuous variables, and the Fisher's exact test for categorical variables. All significant variables were included in the multivariable logistic regression model to predict 1-year survival in the study cohort.

## Results

17,478 patients met the inclusion criteria and were included in the study. 15,449 (88.39%) survived greater than 365 days, whereas 2,029 (11.61%) did not. Variables associated with decreased one-year survival include: age, hospital length of stay, number of hospital admissions post ICU discharge, duration of mechanical ventilation and vasopressor use, a diagnosis of sepsis, history of congestive heart failure (CHF), end-stage renal disease (ESRD), dementia, cirrhosis, cerebro-vascular accident (CVA), chronic obstructive pulmonary disease (COPD), and the need for renal replacement therapy (RRT) or tracheostomy. These results were true for both univariate and multivariate analysis. The following interaction terms were found to be significant: Age*cirrhosis, Age*COPD, Difference in SOFA day_3_-day_1_*sepsis, duration of mechanical ventilation*duration of vasopressor use, duration of mechanical ventilation*cirrhosis, duration of mechanical ventilation*tracheostomy, duration of vasopressor use*cirrhosis, duration of vasopressor use*tracheostomy, ESRD*CHF, RRT*HTN, RRT*sepsis.

## Conclusions

Among critically patients who survive greater than 30 days post discharge, many survive for greater than one year. Factors associated with decreased one-year survival include age, length of stay, number of post-discharge admissions, and numerous co-morbid conditions.

## Grant Acknowledgment

The Laboratory of Computational Physiology receives research funding from the National Institute of Health through Grant R01 EB001659 and Philips.

**Figure 1:**
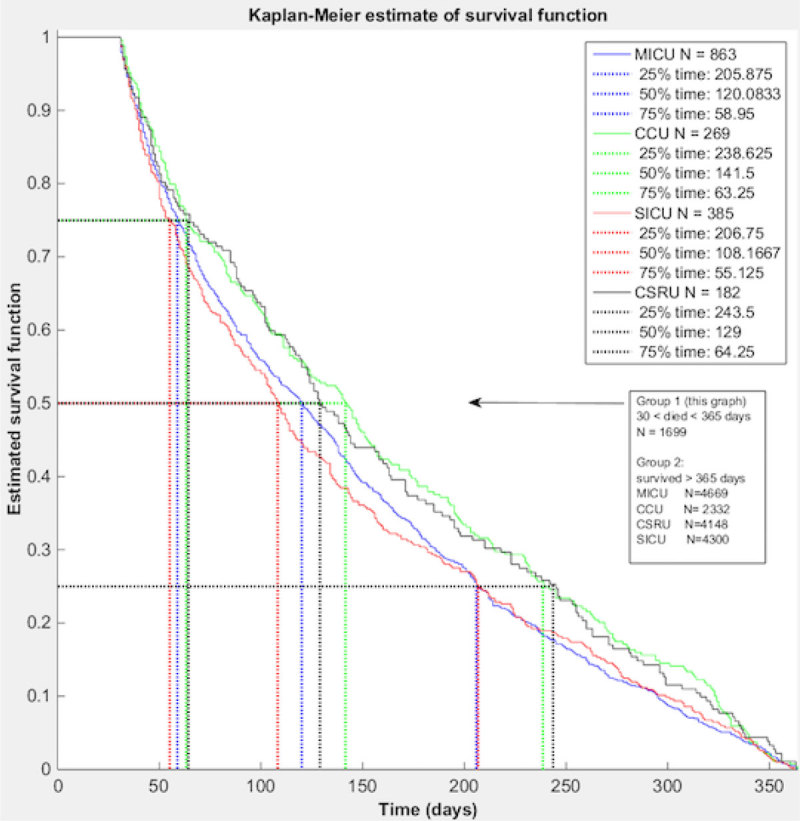
Survival analysis based on type of ICU.

**Table 1:** Comparison based on length of survival.

	30<Survival<365 n = 2029 (11.61%)	Survival>365 n = 15449 (88.39%)	P-value		30<Survival<365 n = 2029 (11.61%)	Survival>365 n = 15449 (88.39%)	P-value
**Age, years**	72.63+14.19	59.83+17.75	0.000	**Hypertension**	1014 (49.98)	7774 (50.32)	0.776
**Congestive Heart Failure**	927 (45.69)	3478 (22.51)	0.000	**Diabetes**	572 (28.19)	3663 (23.71)	0.132
**Dementia**	190 (9.36)	446 (2.89)	0.000	**ICU LOS, days**	2.86 [4.76]	1.99[2.48]	0.000
**Cirrhosis**	157 (7.74)	739 (4.78)	0.000	**Post-discharge Hospital Admissions**	1.21+0.58	1.06+0.28	0.000
**Cerebrovascular Accident**	303 (14.93)	1720 (11.13)	0.000	**SOFA Day 3-Day 1**	1.65	1.25	0.000
**COPD**	554 (27.3)	2575 (16.67)	0.000	**Duration of Mechanical Ventilation, days**	3.73 [12.34]	0.84 [2.29]	0.000
**ESRD**	65 (3.2)	132 (0.85)	0.000	**Duration of Vasopressor Use, days**	1.48 [5.19]	0.77 [1.5]	0.000
**Obesity**	252 (12.42)	3135 (20.29)	0.000	**Renal Replacement Therapy**	147 (8.03)	275 (1.99)	0.000
**Sepsis**	925 (45.59)	2904 (18.8)	0.000	**Acute Kidney Injury**	366 (18.26)	950 (6.33)	0.000

**Table 2:** Multivariable Logistic Regression.

	Odds Ratio	P-value		Odds Ratio	P-value
**Age, years**	1.054	< 0.0001	**Dementia**	1.821	< 0.0001
**Hospital LOS**	1.017	< 0.0001	**Cirrhosis**	7.814	< 0.0001
**Post Discharge Hospital Admissions**	1.761	< 0.0001	**Cerebrovascular Accident**	1.225	0.0055
**SOFA Score Day 3-Day 1**	1.062	< 0.0001	**COPD**	3.220	0.0002
**Duration of Mechanical Ventilation, days**	1.024	0.0002	**Tracheostomy**	2.378	< 0.0001
**Duration of Vasopressor Use, days**	0.965	0.0250	**Obesity**	0.750	0.0001
**ESRD**	6.758	< 0.0001	**Hypertension**	0.669	< 0.0001
**Renal Replacement Therapy**	2.968	< 0.0001	**Sepsis**	1.739	< 0.0001
**Congestive Heart Failure**	1.475	< 0.0001			

**Table 3:** Interaction Terms for Multivariable Regression.

	Odds Ratio	P-value		Odds Ratio	P-value
Age, years * Cirrhosis	0.981	0.0123	Duration of Vasopressor Use * Cirrhosis	1.102	0.0123
Age, years * COPD	0.99	0.0227	Duration of Vasopressor Use * Tracheostomy	1.065	0.0017
Sofa Score Day 3-Day 1 * Sepsis	0.952	0.004	ESRD * Congestive Heart Failure	0.478	0.0292
Duration of Mechanical Ventilation * Duration of Vasopressor Use	1.001	0.0082	Renal Replacement Therapy * Hypertension	2.018	0.0071
Duration of Mechanical Ventilation * Cirrhosis	0.931	0.0032	Renal Replacement Therapy * Sepsis	0.468	0.0017
Duration of Mechanical Ventilation * Tracheostomy	0.953	0.0002			

